# Cross-Cultural Adaptation and Psychometric Properties of the Reward-Based Eating Drive Scale (RED-13) and Its Brief Version (RED-5X) in Three European Countries

**DOI:** 10.3390/nu18010049

**Published:** 2025-12-23

**Authors:** Rui Poínhos, Joanna Kowalkowska, Nicolò Sala, Tainá Lopes da Silva, Marta Plichta, Ana Lucas, Camilla Folzi, Iolanda Cioffi, Ana Maria Pandolfo Feoli, Marisa Porrini, Janete de Souza Urbanetto, Simona Bertoli, Bruno M. P. M. Oliveira

**Affiliations:** 1 Faculty of Nutrition and Food Sciences, University of Porto, Rua do Campo Alegre 823, 4150-180 Porto, Portugal; 2001analucas@gmail.com (A.L.); bmpmo@fcna.up.pt (B.M.P.M.O.); 2 Department of Biology and Environment, School of Life and Environmental Sciences, University of Trás-os-Montes e Alto Douro, Quinta de Prados, 5000-801 Vila Real, Portugal; 3 Department of Human Nutrition, Faculty of Food Science, University of Warmia and Mazury in Olsztyn, Słoneczna 45F, 10-718 Olsztyn, Poland; joanna.kowalkowska@uwm.edu.pl; 4 Faculty of Agricultural and Food Sciences, University of Milan, Celoria 2, 20133 Milan, Italy; 0.nic.sala@gmail.com (N.S.); camilla.folzi@gmail.com (C.F.); 5 Postgraduate Program in Psychology, School of Health and Life Sciences, Pontifical Catholic University of Rio Grande do Sul (PUCRS), Av. Ipiranga 6681, Partenon, Porto Alegre 90619-900, RS, Brazil; nutri.tainalopes@gmail.com (T.L.d.S.); anafeoli@pucrs.br (A.M.P.F.); 6 Department of Food Market and Consumer Research, Institute of Human Nutrition Sciences, Warsaw University of Life Sciences (SGGW-WULS), Nowoursynowska 159c, 02-776 Warsaw, Poland; marta_plichta@sggw.edu.pl; 7 Department of Food, Environmental and Nutritional Sciences (DeFENS), University of Milan, Celoria 2, 20133 Milan, Italy; iolanda.cioffi@unimi.it (I.C.); marisa.porrini@unimi.it (M.P.); 8 Graduate Program in Biomedical Gerontology (GERONBIO), School of Medicine, Pontifical Catholic University of Rio Grande do Sul, Av. Ipiranga, 6681, Partenon, Porto Alegre 90619-900, RS, Brazil; jurbanetto@pucrs.br; 9 Obesity Unit and Laboratory of Nutrition and Obesity Research, Department of Endocrine and Metabolic Diseases, IRCCS Istituto Auxologico Italiano, 20145 Milan, Italy; simona.bertoli@unimi.it; 10 International Center for the Assessment of Nutritional Status and the Development of Dietary Intervention Strategies (ICANS-DIS), Department of Food, Environmental and Nutritional Sciences (DeFENS), University of Milan, Celoria 2, 20133 Milan, Italy; 11 Laboratory of Artificial Intelligence and Decision Support, Institute for Systems and Computer Engineering, Technology and Science (LIIAD, INESC-TEC), 4200-465 Porto, Portugal

**Keywords:** eating behavior, reward-based eating, psychometrics, cross-cultural comparison, validation studies, RED-13, RED-X5

## Abstract

**Background and aims:** Reward-based eating reflects hedonic drivers of intake, including loss of control, diminished satiety, and preoccupation with food. We translated, adapted and studied the psychometric properties of the 13- and 5-item Reward-Based Eating Drive Scale (RED), for Portugal, Poland and Italy. **Methods:** A cross-cultural study was conducted with higher education students and general population samples (n = 1999). After translation and cultural adaptation, the RED was administered with food craving items, and collection of sociodemographic and anthropometric data. Factorial structure and measurement invariance were tested using confirmatory factor analysis (CFA), internal consistency with Cronbach’s alpha, and convergent validity via correlations with BMI and cravings. **Results:** CFA supported the expected structures of the RED-13 (three factors) and RED-X5 (unifactorial), with configural and metric invariance across countries and groups. Only partial scalar invariance was achieved for both versions. The RED-13 showed good to excellent internal consistency for total scores (0.868 ≤ α ≤ 0.906), with acceptable to good reliability for Loss of control (0.769 ≤ α ≤ 0.821), lower values for Lack of satiety (0.655 ≤ α ≤ 0.723), and good to excellent consistency for Preoccupation with food (0.881 ≤ α ≤ 0.918). The RED-X5 showed acceptable internal consistency (0.737 ≤ α ≤ 0.811) and correlated strongly with RED-13 (r = 0.949, *p* < 0.001). Both correlated positively with BMI and food cravings. Age, sex, and country had small to medium multivariate effects on RED scores. **Conclusions:** The RED-13 and RED-X5 showed good psychometric properties in Portugal, Poland, and Italy, with the RED-13 providing a multifactorial assessment and the RED-X5 offering a brief alternative.

## 1. Introduction

The global rise in overweight and obesity has highlighted maladaptive eating behaviors shaped by biological, psychological, and socio-cultural factors [1,2]. Research emphasizes hedonic processes, in which food is consumed for pleasure rather than physiological necessity [2,3], leading to overeating, particularly of foods high in sugar, fat, and salt [1,3,4,5]. These foods activate the brain’s reward circuitry, which plays a central role in motivation, reinforcement, and the experience of pleasure. Repeated exposure can reduce reward sensitivity, promoting compulsive intake similar to substance use disorders [2,5,6,7], particularly in environments where energy-dense, ultra-processed foods are ubiquitous, affordable, and aggressively marketed, conditions that foster reward-driven eating and challenge efforts toward dietary regulation [1,7,8].

The construct of reward-based eating captures behavioral patterns such as loss of control over food intake, reduced sensitivity to satiety signals, and persistent preoccupation with food-related thoughts and cravings [5,9,10,11]. Measuring reward-based eating allows to explain individual susceptibility to overeating and guide tailored interventions [1,6,7,10,11].

The Reward-Based Eating Drive (RED) scale was developed to fill a gap in the assessment of eating behavior by providing a focused measure of reward-based eating tendencies, which are distinct from general measures of disinhibition or emotional eating. It was developed as a 9-item measure (RED-9), assessing three dimensions (factors) of reward-driven eating: (1) Loss of control over eating, reflecting difficulties regulating food intake; (2) Lack of satiety, indicating impaired recognition of fullness; and (3) Preoccupation with food, measuring persistent thoughts, concerns and cravings related to food and eating [10].

The RED-9 demonstrated good reliability and predictive validity, particularly in relation to body mass index (BMI) and weight gain over time [10]. However, subsequent research identified limitations in its psychometric performance, which motivated the development of a revised 13-item version (RED-13), which expanded item coverage through item response theory methods to improve measurement precision, particularly at lower levels of the construct [11]. The RED-13 retained the same three-factor structure while offering enhanced internal consistency and model fit.

More recently, a brief 5-item version (RED-X5) was developed to provide a rapid, unidimensional screening tool [12]. The RED-X5 was constructed by selecting items from the RED-13 with the strongest factor loadings and widest distributional properties, ensuring conceptual breadth. This version does not differentiate subdomains but has demonstrated good internal consistency and strong convergent validity with BMI and food craving measures. Importantly, the RED-X5 is strongly correlated with the RED-13 total score, supporting its utility as a valid proxy for the full scale.

To date, the RED scale has been adapted and validated in a limited number of languages and countries. In 2023, the RED-13 was translated and culturally adapted for Brazilian Portuguese [13]. More recently, Yavuz et al. (2025) published the validation study of the Turkish version of the RED-13 [14]. Additionally, a Spanish translation of the RED-13 is being implemented within larger protocols of two randomized controlled trials [15,16] and a German version of the RED-9 and RED-13 have been developed and preliminarily evaluated in a preprint [17]. To the best of our knowledge, no other language adaptations or validations have been published, nor has the brief 5-item version (RED-X5) been subjected to psychometric evaluation in any population to date. This highlights the need for further cross-cultural validation studies, including of the abbreviated scale, to facilitate its wider application in diverse settings.

While previous studies have typically been conducted within individual countries, conducting psychometric analyses across multiple countries concurrently is essential to assess measurement invariance and ensure that the instrument captures the same underlying construct across diverse cultural and linguistic contexts [18,19]. This approach strengthens the comparability of findings, enhances the generalizability of the scale, and supports its use in multinational research and clinical applications. In this context, it becomes equally important to consider not only linguistic translation but also cultural and regional adaptation. For instance, although a Brazilian Portuguese version of the RED-13 has been developed [13], it reflects the linguistic, cultural, and contextual characteristics of Brazil. Brazilian Portuguese differs meaningfully from European Portuguese, underscoring the need for a separate adaptation for use in Portugal to ensure linguistic and cultural accuracy.

Importantly, to date, no study has conducted a simultaneous cross-national adaptation and psychometric evaluation of the RED scale across multiple European countries. Examining the scale across different languages and cultural contexts provides a particularly stringent test of its cross-cultural robustness, especially given cross-national differences in eating behaviours that may be reflected in reward-driven responses to food. Such an approach allows for a more rigorous assessment of measurement equivalence and supports meaningful cross-country comparisons in both research and applied settings.

To address the current gap in cross-cultural research on reward-based eating, the present study aimed to conduct a comprehensive cross-national validation of the RED scale. By simultaneously adapting and evaluating the RED-13 and RED-X5 in three European countries—Portugal, Poland, and Italy—this work assesses the scale’s psychometric performance across diverse linguistic and cultural contexts. The specific aims of this study were: (1) To translate and culturally adapt the RED scale into European Portuguese, Polish, and Italian; (2) To evaluate the factorial structure of both the RED-13 and RED-X5 versions through confirmatory factor analysis, and to examine measurement invariance (configural, metric, and scalar) across countries and demographic subgroups (higher education students vs. general population); (3) To assess the internal consistency of the RED-13 (total scale and subscales) and the RED-X5; (4) To examine convergent validity by analyzing the associations of RED scores with BMI and with savoury and sweet food cravings; (5) To evaluate the association between the short form (RED-X5) and the full form (RED-13) total scores; and (6) To analyze the multivariate effects of country, group, sex, and age on RED-13 total scores, RED-13 subscale scores, and RED-X5 scores.

## 2. Methods

### 2.1. Study Design and Ethics

This was a cross-sectional, cross-cultural methodological study conducted simultaneously in Portugal, Poland and Italy. The study was structured into two main phases:

Phase 1 focused on the translation and cultural adaptation of the RED. Additionally, a set of eight craving-related items was also translated and culturally adapted during this phase, using a simplified version of the procedure applied to the RED. These items were intended to be used for the validation of the RED through convergent analysis, replicating procedures used in prior RED validation studies [11,12,13].

Phase 2 involved the administration of an online questionnaire containing the translated RED versions and responses to measures of cravings for sweet and savory foods, along with sociodemographic questions and self-reported weight and height. Data collection took place between April 2024 and May 2025. This phase aimed to evaluate and compare the psychometric properties of the RED-13 and RED-X5 across countries and population groups.

The study protocol was submitted to and approved by the Ethics Committee of the Faculty of Nutrition and Food Sciences of the University of Porto (rapport number 133/2023) and by the Data Protection Unit of the University of Porto (approval A-20/2024). All participants were informed about the study’s objectives and procedures and provided informed consent prior to participation. No personally identifiable information was collected. Permission to translate and adapt the original scale was formally obtained from its authors.

### 2.2. Phase 1: Translation and Cultural Adaptation

The translation and cultural adaptation were conducted simultaneously in the three countries: Portugal (European Portuguese), Italy (Italian), and Poland (Polish). The process was guided by the methodological principles proposed by Eremenco et al. (2018) [20] for the cross-cultural adaptation of self-report instruments.

In each country, the adaptation began with three independent forward translations performed by native-speaking translators, for whom knowledge of psychometrics and nutritional psychology was ensured through shared methodological guidelines and standardized, pre-aligned translation procedures. These were subsequently reconciled into a single forward translation by consensus among the respective national translation teams. In Portugal, the existing Brazilian Portuguese version of the RED was also taken into consideration after the reconciliation process; however, this comparison did not result in any modifications to the reconciled European Portuguese version. In each country, a back-translation of the reconciled version was then conducted by an independent translator with proficiency in English and no prior exposure to the original instrument.

The original English version and the back-translated version were reviewed and compared by the original translators to assess semantic equivalence and identify potential discrepancies. Discrepancies were either (a) justified by linguistic or cultural considerations or (b) used to revise the reconciled version accordingly. This process was informed by the universalist approach to equivalence outlined by Herdman et al. (1998) [21].

As part of the international harmonization phase, the translation teams from the three countries collaboratively reviewed all versions to ensure cross-linguistic consistency and conceptual equivalence across languages and with the original instrument. Based on this review, appropriate linguistic adjustments were proposed to ensure cultural and regional relevance. These modifications underwent reconciliation, back-translation, and evaluation to maintain fidelity to the original content. Following harmonization, the pre-final versions in each country underwent proofreading by two independent native speakers to correct any remaining orthographic, grammatical, or typographical issues.

Finally, cognitive interviews and pilot testing were conducted in person in each country with at least five participants per language version. Participants were asked to verbalize their thought process while responding to each item, ensuring that questions were interpreted as intended and that responses accurately reflected their lived experience. Feedback from these interviews was reviewed by the translation teams and informed final revisions to the instrument.

### 2.3. Phase 2: Sample and Recruitment

Data were collected from two samples in each participating country: one comprising higher education students and the other comprising members of the general adult population. Eligibility criteria were identical across samples and countries, requiring participants to be 18 years of age or older, with no additional inclusion or exclusion criteria applied. To define the minimum sample size, a minimum participant-to-item ratio of 10:1 was adopted [22]. As the RED-13 includes 13 items, this resulted in a minimum of 130 participants per subsample.

Higher education students were invited to participate via institutional email lists, internal communication platforms, or in person in the classroom setting. Members of the general population were recruited primarily through announcements on social media and online networks. Data collection was conducted online through LimeSurvey, hosted on the University of Porto’s survey platform.

### 2.4. Phase 2: Instruments

The online questionnaire began with a description of the study’s aims and procedures and a request for informed consent. The first section included questions on the participants sex (female/male), age (years), and self-reported weight (kg) and height (cm). BMI (kg/m^2^) was calculated for each participant from the reported weight and height.

In the second section, participants answered eight craving-related items, adapted from the Control of Eating Questionnaire (CoEQ) [23]. Participants were instructed to respond based on their experiences “over the last seven days.” Four items assessed cravings for savoury foods (e.g., “How strong was your desire to eat savoury foods?”), and four items assessed cravings for sweet foods (e.g., How often have you had cravings for chocolate and chocolate flavoured foods?). All items were rated on scale ranging from 1 (“not at all strong”/“not at all”) to 100 (“extremely strong”/“extremely often”). Total scores for each subscale (sweet, savoury) were computed as the mean of items for that subscale, with higher scores indicating stronger or more frequent cravings.

The third section comprised the translated RED-13 scale, which assesses three dimensions of reward-driven eating: Loss of control (items 1, 2, 3, 4, 12 and 13), Lack of satiety (items 5, 6 and 10), and Preoccupation with food (items 7, 8, 9 and 11). Participants responded on a 5-point Likert scale ranging from 0 (strongly disagree) to 4 (strongly agree). Scores for the RED-13 total scale, its three dimensions, and the shorter RED-5X version (items 2, 4, 6, 8, and 9) were calculated as the mean of their respective items, with higher scores indicating greater reward-based eating.

### 2.5. Phase 2: Statistical Analysis

Statistical analyses were conducted using IBM SPSS Statistics for Windows, version 30.0. Descriptive statistics included absolute and relative frequencies (n, %), means and standard deviations for age, and median and interquartile range (P25, P75) for BMI, as BMI was not normally distributed (based on skewness and kurtosis). All inferential analyses were performed with a 95% confidence level.

Confirmatory Factor Analysis (CFA) was conducted using IBM SPSS AMOS 29. It was not possible to achieve the minimum sample size of 130 higher education students in Italy, and so the two Italian subsamples were not analyzed separately in the confirmatory factor analysis. Throughout the analysis, only modification indices (MI) greater than 10 were considered when adjusting the model to improve fit [24].

The analysis followed a stepwise procedure assessing configural, metric, and scalar invariance [18,19]. Model fit was evaluated using χ^2^, NFI, RFI, TLI, CFI, RMSEA, and SRMR, based on commonly accepted guidelines [25]. A χ^2^/*df* ratio ≤ 3 indicated acceptable fit, but due to the large sample size, χ^2^ difference tests were expected to be significant. CFI and TLI values ≥ 0.90 were considered adequate and ≥0.95 as indicative of good fit; RMSEA values ≤ 0.08 reflected reasonable error of approximation and ≤0.05 a close fit; and SRMR values ≤ 0.08 were considered acceptable.

Invariance testing focused on changes in fit indices between nested models. Changes in fit indices (using values reported by the software, which may not exactly match the simple arithmetic differences between absolute values) were evaluated according to commonly accepted criteria. Specifically, decreases in NFI, RFI, TLI or CFI less than or equal to 0.010 and increases in RMSEA less than or equal to 0.015 indicate acceptable measurement invariance [26].

Latent mean comparisons across groups were conducted within the partial scalar invariance framework established in the previous steps. Latent means for the reference group (Portuguese general population, the group with larger sample size) were fixed at zero, while latent means for the other groups were freely estimated.

Cronbach’s alpha coefficients were calculated to assess the internal consistency of the RED-13 total score, its three dimensions, and the RED-X5. Internal consistency was considered excellent if α ≥ 0.9, good if α ∈ [0.8; 0.9[, acceptable if α ∈ [0.7; 0.8[, questionable if α ∈ [0.6; 0.7[, poor if α ∈ [0.5; 0.6[ and unacceptable if α < 0.5 [27].

Convergent validity was assessed by examining correlations between RED scores and participants’ BMI (Spearman’s correlation coefficient, rs) and craving scores (Pearson’s correlation coefficient, r). Correlations were classified as very weak if |r| or |rs| < 0.25, weak if |r| or |rs| ∈ [0.25; 0.5[, moderate if |r| or |rs| ∈ [0.5; 0.75[, strong if |r| or |rs| ∈ [0.75; 0.9[ and very strong if |r| or |rs| ∈ [0.9; 1] [28].

MANCOVA and ANCOVA models were run to analyse the effects of sex (female/male), age (years), country (Portugal/Poland/Italy), and group (higher education students/general population) on RED scores. In the MANCOVA, the three RED-13 factors were entered as dependent variables. Two separate ANCOVAs were conducted: one using the RED-13 total score and another using the RED-X5 score as the dependent variable. Effect sizes were quantified using partial eta-squared (η_p_^2^) and interpreted as small (η_p_^2^ < 0.030), medium (0.030 ≤ η_p_^2^ < 0.100), or large (η_p_^2^ ≥ 0.100) based on the qualitative definition of between-subject effects originally proposed by Cohen (1988) [29].

## 3. Results

### 3.1. Phase 1: Translation and Cultural Adaptation

Within each national team, consensus was achieved among the forward translators, without the need for external arbitration. When comparing back-translations with the original English version, all discrepancies were deemed to reflect intentional adaptations motivated by cultural or linguistic appropriateness or were semantically equivalent to the original phrasing. Most differences observed in the international harmonization phase had already been justified during translation, primarily reflecting adaptations to ensure natural and culturally appropriate wording, including adjustments in formality or phrasing. A small number of exceptions required further discussion; these involved the scale’s name, instructions, and items 1, 11, and 13.

The Italian team considered that translating the instrument’s full name resulted in an unnatural expression in Italian, and opted to retain the abbreviated form “Scala RED,” followed by the original English name in parentheses. The Portuguese and Polish versions retained the translated full name corresponding to the original English title.

The Polish team proposed changing “question” to “statement” in the instructions, as the items refer to statements rather than questions. This revision was adopted across all versions. In the Italian version of the instructions, the initial translation included two additional expressions: “utilizzando la seguente scala” (“using the following scale”) and the possessive “vostro” (“your”) in “vostro livello di accordo” (“your level of agreement”). These additions were removed following team consensus, as they were considered unnecessary elaborations that did not sufficiently justify a deviation from the original phrasing.

Item 1 (I feel out of control in the presence of delicious food) was rendered in Polish as “I cannot control myself,” due to the absence of a natural equivalent for “I feel out of control.” The Polish version was retained, as it was considered semantically consistent with the original. Item 11 (I can’t stop thinking about eating no matter how hard I try) had back-translations in Italian and Polish that rendered “eating” as “food”. In Polish, this was due to the similarity between the words for “eating” and “food”, while in Italian, the use of “eating” in this context was considered unnatural. In both cases, the wording was judged to be semantically equivalent, and the translations were therefore maintained. The Portuguese back-translation matched the original item exactly. Item 13 (If food tastes good to me, I eat more than usual) had the Italian back-translation “If I like food” instead of “If food tastes good to me,” reflecting a syntactic reordering. As no substantial deviation in meaning was identified, the phrasing was retained.

Cognitive interviews were conducted with six participants per country (three females, three males; heterogeneous in age). Although overall comprehension was good across countries, a few minor issues were identified. The Italian interviews did not reveal any significant comprehension difficulties. In Poland, the wording of Item 13 was slightly rephrased to improve clarity through a change in word order. Item 3 (It is difficult for me to leave food on my plate) prompted interpretive comments in Portugal and Poland, where some participants associated the item with concerns about food waste. In Portugal, Item 4 (When it comes to foods I love, I have no willpower) raised questions about the phrasing of “willpower”, as participants noted that the Portuguese expression “força de vontade” typically requires contextual specification (e.g., willpower to do something). Despite these observations, participants in all countries ultimately understood and responded to the items as intended. No changes were made to Items 3 and 4 to avoid redundancy with other items and preserve fidelity to the original scale. The final versions of the scale are available as Supplementary Material (Table S1).

### 3.2. Phase 2: Psychometric Characteristics and Group Comparisons

Table 1 presents descriptive information on the participants by country (Portugal, Poland, Italy) and group (higher education students vs. general population).

#### 3.2.1. Confirmatory Factor Analysis and Latent Mean Comparison of RED-13

Initial evaluation of the model fit indices revealed suboptimal fit in some groups, prompting an examination of modification indices to identify potential sources of misfit. Based on the highest modification indices, correlations between error terms were added to improve model fit. A residual covariance was included between items 12 and 13, which belong to the same latent factor (Factor 1). Additionally, residual covariances were allowed between items 3 (“It is difficult for me to leave food on my plate”, Factor 1) and 6 (“I don’t get full easily”, Factor 2), and between items 10 (“I feel hungry all the time”, Factor 2) and 11 (“I can’t stop thinking about eating no matter how hard I try”, Factor 3), as each pair reflects overlapping content not fully captured by the latent constructs—specifically behavioral and physiological aspects of eating persistence, and a combination of physiological hunger with cognitive preoccupation. These adjustments improved overall fit without altering the theoretical factor structure.

**Figure 1:**
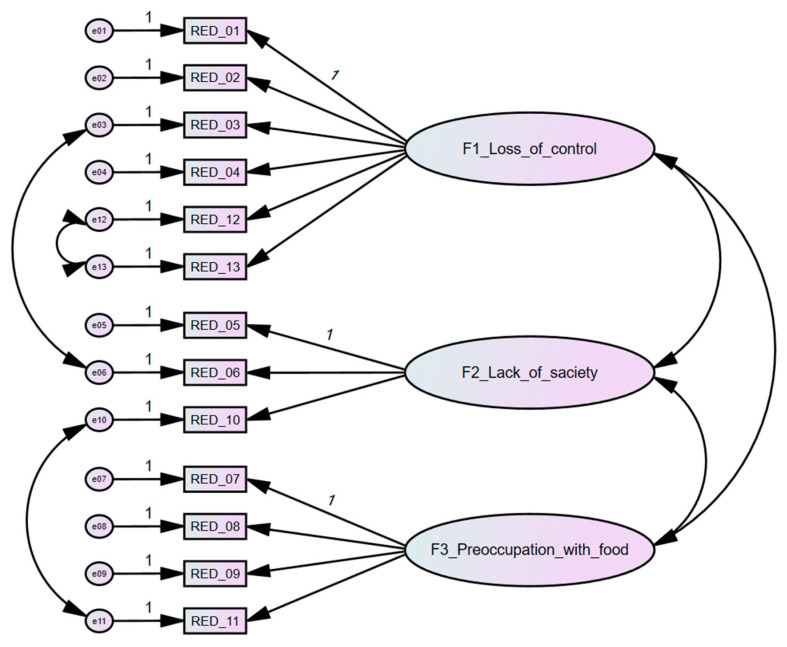
Configural model of RED-13 with specified error covariances.

Using this adjusted model, configural invariance was tested across the five groups to assess whether the factorial structure, including the specified residual covariances, was acceptable without imposing equality constraints on parameters (Table 2). The configural model demonstrated adequate fit across groups (χ^2^ = 828.066, *df* = 295, *p* < 0.001, NFI = 0.935, RFI = 0.914, TLI = 0.943, CFI = 0.957, RMSEA = 0.030), indicating that the RED-13 scale exhibits a consistent factorial structure among the different samples. Therefore, the model (Figure 1) was retained without further modifications.

Next, metric invariance was tested by comparing the configural model to the metric model (with factor loadings constrained equal across groups). Although the chi-square difference test was statistically significant (Δχ^2^ = 83.638, Δ*df* = 40, *p* < 0.001), likely due to the large sample size, the metric invariance model showed an acceptable fit, with minimal changes in fit indices: ΔCFI = 0.007, ΔNFI = 0.007, ΔRFI = −0.003, and ΔTLI = −0.003. These differences fall well below the recommended threshold of 0.01 [26], indicating that constraining factor loadings to be equal across groups did not substantially worsen model fit. Overall, the evidence supports metric invariance of the RED-13 scale across the five groups (Table 2).

However, scalar invariance was not achieved, as constraining the item intercepts to equality significantly worsened model fit: Δχ^2^ = 724.274, Δ*df* = 52, *p* < 0.001, ΔCFI = 0.058, ΔNFI = 0.057, ΔRFI = −0.046, and ΔTLI = 0.048. Since full scalar invariance was not achieved, intercepts with modification indices greater than 10 [24] were freed iteratively, starting from the largest MI and proceeding one at a time, reassessing model fit after each step. A total of 18 intercepts (out of 65) were freed, resulting in a partial scalar invariance model with substantially improved fit (Table 2). The distribution of freed intercepts varied across factors and groups. Factor 2 showed the highest number of freed intercepts relative to its number of items (six intercepts across three items), followed by Factor 1 (nine intercepts across six items), whereas Factor 3 required the fewest freed intercepts (three intercepts across four items). Across groups, the number of freed intercepts was highest in the Italian sample (n = 5), followed by both Polish samples (4 each), the Portuguese general population sample (n = 3), and the Portuguese student sample (n = 2).

Compared to the metric invariance model, NFI decreased slightly from 0.928 to 0.918 (ΔNFI = 0.011), RFI from 0.917 to 0.913 (ΔRFI = 0.004), TLI from 0.946 to 0.942 (ΔTLI = 0.004), and CFI from 0.953 to 0.945 (ΔCFI = 0.011). RMSEA remained low, changing marginally from 0.029 to 0.030 (90% CI [0.028, 0.033]). These variations fall within commonly accepted thresholds for measurement invariance testing [26], supporting that the RED-13 scale functions comparably across groups, allowing valid latent mean comparisons despite some intercept differences.

Using the partial scalar invariance model, latent means were compared across the five groups in a multigroup framework, with the Portuguese general population group as the reference (latent means fixed at zero; Table 3). The Portuguese students group showed no significant differences from the reference group across all three factors (F1 Estimate = 0.039, *p* = 0.589; F2 Estimate = 0.033, *p* = 0.611; F3 Estimate = 0.084, *p* = 0.277). In contrast, the Polish students and Italy groups exhibited significantly higher latent means on all factors (Polish students: F1 = 0.286, *p* < 0.001; F2 = 0.173, *p* = 0.003; F3 = 0.416, *p* < 0.001; Italy: F1 = 0.230, *p* < 0.001; F2 = 0.435, *p* < 0.001; F3 = 0.252, *p* < 0.001). The Polish general population group displayed a higher mean on Factor 1 (Estimate = 0.164, *p* = 0.007) but significantly lower means on Factors 2 (Estimate = −0.219, *p* < 0.001) and 3 (Estimate = −0.171, *p* = 0.004) compared to the reference group.

#### 3.2.2. Confirmatory Factor Analysis and Latent Mean Comparison of RED-X5

Initial evaluation of the model fit indices revealed suboptimal fit, prompting an examination of modification indices to identify potential sources of misfit. Based on the highest modification indices, residual correlations were added between items 2 (“When I start eating, I just can’t seem to stop”) and 4 (“When it comes to foods I love, I have no willpower”), between items 2 and 6 (“I don’t get full easily”), and between items 4 and 6. These residual correlations reflect shared item-specific variance, likely due to similarities in content or wording, which are not captured by the single latent factor. These adjustments improved model fit without compromising the interpretation of the total score.

**Figure 2:**
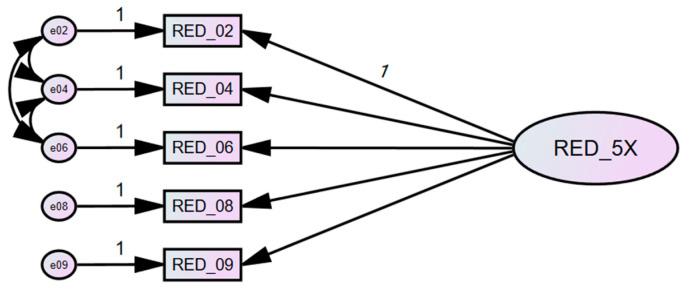
Configural model of RED-X5 with specified error covariances.

Using this adjusted model, configural invariance was tested across the five groups to assess whether the factorial structure, including the specified residual covariances, was acceptable without imposing equality constraints on parameters. The configural model demonstrated adequate fit across groups (χ^2^ = 20.794, *df* = 10, *p* = 0.023, NFI = 0.994, RFI = 0.968, TLI = 0.983, CFI = 0.997, RMSEA = 0.023), indicating that the unifactorial structure of the RED-5X fits well across all groups (Table 4). Therefore, the model (Figure 2) was retained without further modifications.

Metric invariance was tested by comparing the configural model to the metric model. Although the chi-square difference test was statistically significant (Δχ^2^ = 27.843, Δ*df* = 16, *p* = 0.033), the metric invariance model showed an acceptable fit, with minimal changes in fit indices (Table 4): ΔCFI = 0.008, ΔNFI = 0.008, ΔRFI = −0.003, and ΔTLI = −0.003. These differences fall below the threshold of 0.01 [26], indicating that constraining factor loadings to be equal across groups did not substantially worsen model fit. Overall, the evidence supports metric invariance of the RED-5X scale across the five groups.

However, scalar invariance was not achieved, as constraining the item intercepts to equality significantly worsened model fit: Δχ^2^ = 364.546, Δ*df* = 20, *p* < 0.001, ΔCFI = 0.111, ΔNFI = 0.110, ΔRFI = 0.108, and ΔTLI = 0.109. Since full scalar invariance was not achieved, intercepts with modification indices greater than 10 [24] were freed iteratively, starting from the largest MI and proceeding one at a time, reassessing model fit after each step. A total of 7 intercepts (out of 25) were freed, resulting in a partial scalar invariance model with substantially improved fit (Table 4). The number of freed intercepts was highest in the Polish samples (2 in each), whereas one intercept was freed in each of the remaining groups (Italian, Portuguese students, and Portuguese general population).

Compared to the metric invariance model, NFI decreased from 0.985 to 0.969 (ΔNFI = 0.016), RFI from 0.972 to 0.961 (ΔRFI = 0.011), TLI from 0.987 to 0.975 (ΔTLI = 0.011), and CFI from 0.993 to 0.981 (ΔCFI = 0.016). RMSEA increased slightly from 0.021 to 0.028 (90% CI [0.022, 0.035]). Although the changes in CFI and TLI slightly exceeded the commonly recommended cutoff of 0.010 [26], the absolute fit indices remained high, all within ranges considered indicative of good model fit. Overall, these results support partial scalar invariance for the RED-5X scale, suggesting that despite some intercept differences, the scale functions comparably across groups and allows for meaningful latent mean comparisons.

Using the partial scalar invariance model, latent means were compared across the five groups in a multigroup framework, with the Portuguese general population group serving as the reference (latent mean fixed at zero; Table 5). The Portuguese students’ group (Estimate = 0.050, *p* = 0.249) and the Polish general population group (Estimate = −0.046, *p* = 0.185) showed no significant differences from the reference group. Both the Polish students’ group (Estimate = 0.163, *p* < 0.001) and the Italian sample (Estimate = 0.105, *p* = 0.009) presented significantly higher latent means compared to the reference.

#### 3.2.3. Internal Consistency and Convergent Validity

Internal consistency was assessed using Cronbach’s alpha coefficients for the RED-13 total scale, its three subscales, and the short form RED-X5, across all six subsamples (Table 6). The RED-13 total scale demonstrated good to excellent internal consistency in all groups, with alpha values ranging from 0.868 (Portuguese students) to 0.906 (Portuguese general population). Among the subscales, Loss of Control (Factor 1) showed acceptable to good internal consistency, with alpha values ranging from 0.769 to 0.821 across groups. Lack of satiety (Factor 2) yielded lower reliability, with alpha values between 0.655 and 0.723, indicating questionable to acceptable internal consistency. Preoccupation with food (Factor 3) showed good to excellent internal consistency, with alpha values from 0.881 to 0.918. The short form, RED-X5, demonstrated acceptable internal consistency across all samples, with Cronbach’s alpha ranging from 0.737 (Polish students) to 0.811 (Italian students). For descriptive purposes, we also present the overall means and standard deviations for the RED-13 total, subscales and RED-X5.

To examine convergent validity, correlations were computed between RED scores (total and subscale scores of the RED-13, and the total score of the RED-X5) and variables theoretically linked to reward-based eating (BMI and food cravings for savoury and sweet foods). As presented in Table 7, all correlations were weak or very weak, but all RED scores were positively and significantly correlated with BMI and both craving indices, except for the Preoccupation with food subscale (Factor 3), which showed a non-significant correlation with BMI (r = 0.043, *p* = 0.053). The highest association was found for the RED-13 total score with savoury cravings (r = 0.315, *p* < 0.001), and, overall, correlations with savoury cravings were higher than those observed with BMI and sweet cravings. Among the RED-13 subscales, the strongest associations were observed for the Loss of Control subscale (Factor 1).

The same correlations were computed stratified by country, group, and sex. Overall, the pattern of weak or very weak positive associations with BMI and food cravings was consistent across all subsamples. Not focusing on differences in statistical significance, which are influenced by sample size, some specific patterns emerged. Correlations tended to be higher overall in the Portuguese subsamples compared to Poland and Italy. The stronger correlations with savoury cravings, relative to BMI and sweet cravings, were particularly pronounced among Portuguese male students. Among Portuguese males from the general population and Italian male students, correlations with food cravings (with little distinction between savoury and sweet cravings) were higher than those observed with BMI. Detailed results for all subsamples are available as Supplementary Material (Table S2).

Additionally, we examined the association between the RED-X5 and RED-13 total scores. The correlation was very strong in the overall sample (n = 1999; r = 0.949, *p* < 0.001), as well as across the six subsamples defined by country (Portugal, Poland, Italy) and participant type (students vs. general population), with correlations ranging from r = 0.940 to r = 0.957 (all *p* < 0.001).

#### 3.2.4. Multivariate Effects Country, Group, Sex and Age on RED Scores

Table 8 presents the results of the ANCOVA model used to analyse the effects of country, group, sex, and age on RED-13 total scores. The model was statistically significant (*p* < 0.001) and explained 5.3% of the variance in RED-13 total scores (adjusted R^2^ = 0.053). Among the independent variables, country, sex, and age showed significant effects, although all effect sizes were small. Age had the strongest effect: higher age was associated with lower reward-based eating tendencies (β = −0.010, *p* < 0.001, η_p_^2^ = 0.026). Regarding country differences (*p* = 0.009, η_p_^2^ = 0.005), Italian participants had significantly higher RED-13 total scores than Portuguese participants (β = 0.137, *p* = 0.004, η_p_^2^ = 0.004). Males reported significantly lower RED-13 total scores compared to females (β = −0.069, *p* = 0.045, η_p_^2^ = 0.002). No significant effect was found for group (general population vs. students, *p* = 0.697, η_p_^2^ < 0.001).

Table 9 presents the results of the MANCOVA model examining the effects of country, group, sex, and age on the three RED-13 subscales. The overall multivariate model was statistically significant (*p* < 0.001) and explained 2.0% of the variance in Loss of control (Factor 1), 5.6% in Lack of satiety (Factor 2), and 8.9% in Preoccupation with food (Factor 3). Age showed significant negative associations with all three subscales (all *p* < 0.001), with the strongest effect observed for preoccupation with food (β = −0.014, *p* < 0.001, η_p_^2^ = 0.032, medium effect). Males reported significantly lower scores on Preoccupation with food (β = −0.314, *p* < 0.001, η_p_^2^ = 0.026, small effect) and higher scores on Lack of satiety (β = 0.086, *p* = 0.026, η_p_^2^ = 0.002, small effect). Regarding country, Italian participants reported significantly higher scores on Lack of satiety compared to Portuguese participants (β = 0.263, *p* < 0.001, η_p_^2^ = 0.012, small effect), while Polish participants reported lower scores (β = −0.137, *p* = 0.001, η_p_^2^ = 0.005, small effect). No significant effect of group (general population vs. students) was found for any of the RED-13 subscales.

Finally, the results of the ANCOVA model used to analyse the effects of country, group, sex, and age on RED-X5 total scores are presented in Table 10. The model was statistically significant (*p* < 0.001) and explained 4.2% of the variance. No significant effects were found for country (*p* = 0.053, η_p_^2^ = 0.003) or group (general population vs. students, *p* = 0.519, η_p_^2^ = 0.001). Age showed the strongest effect, with higher age being associated with lower RED-X5 scores (β = −0.010, *p* < 0.001, η_p_^2^ = 0.020, small effect). Male participants reported significantly lower RED-X5 scores than females (β = −0.128, *p* < 0.001, η_p_^2^ = 0.006, small effect).

## 4. Discussion

This study aimed to translate, culturally adapt, and study the psychometric properties of the Reward-Based Eating Drive Scale (RED-13 and RED-X5) across three European countries: Portugal, Poland, and Italy. We examined factorial validity, measurement invariance, reliability, and convergent validity in both higher education students and the general population.

### 4.1. Translation and Cultural Adaptation

The translation and cultural adaptation process followed key principles of internationally accepted guidelines for cross-cultural adaptation of self-report instruments [20], ensuring linguistic and conceptual equivalence across versions. By conducting parallel translation efforts in three countries and engaging in systematic harmonization, the process ensured both semantic equivalence and cultural appropriateness across languages.

Minor discrepancies identified during the back-translation and harmonization phases mostly reflected differences in linguistic structures or cultural conventions, without altering the intended meaning of the items. Some items required more nuanced linguistic decisions due to the lack of direct equivalents in some languages. These challenges mirror those described in the Brazilian adaptation, particularly regarding the culturally specific connotations of terms like “willpower” and “control” [13].

The cognitive interviews further confirmed that the translated items were generally well understood, while also revealing subtle cultural interpretations, such as the association between item 3 and food waste concerns in Portugal and Poland. These observations reinforce the importance of combining semantic accuracy with cultural resonance [19,20,21], a balance that was maintained by preserving item fidelity in cases where rewording could introduce redundancy or alter the scope of the construct. Taken together, these results support the cross-linguistic and cross-cultural adequacy of the European Portuguese, Polish, and Italian versions of the RED-13.

### 4.2. Factor Structure and Latent Mean Comparisons

The confirmatory factor analyses supported the expected structures of both the RED-13 and RED-X5 across countries and demographic groups. For the RED-13, the originally proposed three-factor model [10,11] showed good fit, consistent with validations in different cultural contexts, namely Brazil [13], Turkey [14] and Germany [17]. The RED-X5 also exhibited satisfactory model fit, reinforcing its structural adequacy as a concise proxy for the full RED-13 [12].

Both versions demonstrated configural and metric invariance across countries (Portugal, Poland, Italy) and population groups (higher education students vs. general population), indicating that the overall structure and factor loadings were comparable. However, full scalar invariance was not achieved. This suggests that, although the constructs are similarly conceptualized and measured across groups, some item intercepts vary, potentially reflecting subtle cultural or linguistic differences in how respondents interpret certain concepts. Nonetheless, partial scalar invariance was established, which allows for latent mean comparisons under constrained conditions [18,19]. It should be stressed that full scalar invariance is required for direct comparisons; therefore, researchers should exercise caution when interpreting cross-cultural mean differences. Future studies may consider further refinement of the scale or testing alternative items to enhance invariance.

Latent mean comparisons revealed group differences in reward-based eating tendencies, even after establishing partial scalar invariance. While Portuguese students did not differ significantly from the general Portuguese population (reference), meaningful differences emerged in the remaining groups. Polish students and Italian participants showed consistently higher latent means across all RED-13 dimensions and on the RED-X5, suggesting a greater endorsement of reward-based eating tendencies in these populations. In contrast, the Polish general population exhibited a more complex pattern, with higher latent means for Loss of control but lower means for Lack of satiety and Preoccupation with food. These findings underscore potential cross-cultural and subgroup variability in the expression and self-perception of reward-driven eating. However, given that full scalar invariance was not achieved, these differences should be interpreted with caution, as they may partly reflect variation in item intercepts rather than true differences in the underlying constructs [18,19,26].

### 4.3. Internal Consistency and Convergent Validity

Both the RED-13 total score and the RED-X5 demonstrated adequate to excellent internal consistency across all subsamples. The RED-13 total scale yielded Cronbach’s alpha coefficients between 0.868 and 0.906, indicating robust reliability. The subscale Loss of control showed acceptable to good reliability (alphas ranging from 0.769 to 0.821); Lack of satiety presented lower values (0.655 to 0.723), but still within the questionable to acceptable range; and Preoccupation with food showed strong consistency (0.881 to 0.918). These results are consistent with those reported in the cross-cultural adaptations of the scale: the Brazilian adaptation reported an alpha of 0.921 for the total score and factor alphas of 0.876 (Loss of control), 0.908 (Lack of satiety) and 0.792 (Preoccupation with food) [13], while the Turkish version documented a total alpha of 0.906 and factor alphas of 0.879 (Loss of control), 0.846 (Lack of satiety) and 0.848 (Preoccupation with food) [14], further supporting the cross-cultural reliability of the scale. The German version of the RED-13 also showed acceptable internal consistency for the total (alpha = 0.84), Loss of control (alpha = 0.77) and Lack of satiety (alpha = 0.82), despite the factor Preoccupation with food presented lower reliability (alpha = 0.54) [17].

It is important to note that lower alpha values in certain subscales—particularly Lack of satiety, which includes only three items—may reflect the known limitation of Cronbach’s alpha with short item sets. In the present study, the lowest internal consistency estimates for this subscale were observed among student samples in Portugal and Poland, with Cronbach’s alpha of 0.655 and 0.674, respectively. Both the Lack of Satiety and the Preoccupation with Food subscales, as well as the RED-X5, comprise a small number of items, which can lead to an underestimation of internal consistency [30,31]. Despite this, the RED-X5 showed acceptable reliability across all groups (alphas ranging from 0.737 to 0.811), comparable to the values obtained in its original development (omega coefficients presented graphically, with Cronbach’s alphas reported to differ by no more than 0.005) [12]. Taken together, these findings provide strong evidence that both the full (RED-13) and short (RED-X5) versions of the RED scale exhibit consistent internal reliability across diverse cultural and demographic groups, including students and the general population.

The pattern of positive associations between RED scores and theoretically related variables, namely BMI and food cravings [3,4,5,7,9,32], provides support for the convergent validity of both the RED-13 and RED-X5. This approach is consistent with previous RED validation studies, which have examined associations with BMI and food cravings [11,12,13].

The strongest associations were found between the RED-13 total score and savoury cravings, particularly within the Portuguese subsamples and more markedly among male students. While this subgroup effect should be interpreted with caution, it may reflect age- and sex-related differences in responsiveness to palatable food cues. Specifically, younger age is associated with greater neurobiological sensitivity to reward and related behavioral tendencies (e.g., responsiveness to cues of palatable foods) [33,34,35,36]. Also, evidence suggests that men may report stronger cravings for savoury foods relative to women, whose cravings tend to be more focused on sweet items [37].

In contrast, correlations with BMI, although statistically significant, were generally weaker and more variable across subsamples. This finding aligns with the understanding that reward-based eating may contribute to body weight status but does not fully account for it, given that body weight is influenced by a complex interplay of genetic, metabolic, behavioral, and environmental factors [1,2]. The relatively weaker correlations with BMI observed in the present study are consistent with results from the Brazilian validation of the RED-13, where the association with BMI (rs = 0.266) was weaker than those with savoury and sweet cravings (rs = 0.390 and 0.437, respectively) [13]. Similarly, the original development study of the RED-13 reported a weaker correlation with BMI (r = 0.25) compared to savoury cravings (r = 0.47), though the correlation with sweet cravings was of comparable magnitude (r = 0.29) [11]. By contrast, in the original development and validation study of the RED-5X, correlations with sweet cravings were lower than those observed for BMI and savoury cravings, which exhibited comparable strengths [12]. These results highlight the complexity of the relationships between reward-based eating, cravings and weight status, with the relative strength of associations varying across different samples and measures.

Among the RED-13 subscales, Loss of Control consistently showed stronger associations with both food cravings and BMI than Lack of satiety or Preoccupation with food. This pattern is theoretically expected, as Loss of control captures impulsive and compulsive aspects of eating behavior that are central to dysregulated eating and weight-related outcomes [9,10,11,32,38]. While Preoccupation with food may be conceptually more closely linked to the cognitive dimension of craving, especially intrusive thoughts about food [10,11,32], this was not reflected in stronger empirical associations. These findings suggest that Loss of control may represent the most behaviorally proximal expression of reward-based eating, and therefore the most sensitive to individual differences in eating dysregulation and its correlates.

Overall, although the effect sizes are small, these results align with the multifactorial and multidimensional nature of reward-based eating. They highlight the RED-13′s conceptual distinctiveness and its differentiated associations with related constructs, supporting its value in capturing a specific facet of eating behavior not entirely reducible to more commonly measured variables.

While the RED-13 provides a multidimensional profile of reward-based eating, the RED-X5 was designed as a unidimensional short form. Despite not capturing the three distinct dimensions of the full scale, the RED-X5 demonstrated a very strong correlation with the RED-13 total score across the full sample and all subgroups (r ≥ 0.940). This supports the short form’s robustness as a brief alternative to the full scale. These findings are consistent with the original development study of the RED-X5 [12], reinforcing its utility for settings where brevity is essential without compromising psychometric quality.

### 4.4. Multivariate Effects Country, Group, Sex and Age on RED Scores

The multivariate analyses using observed RED scores (via ANCOVA and MANCOVA models) revealed patterns that somewhat align with the findings from the latent mean comparisons, while also offering additional insights into the influence of individual sociodemographic variables. Although effect sizes were generally small, the explained variance differed across the RED-13 dimensions: the highest proportion was observed for Preoccupation with food (8.9%), followed by Lack of satiety (5.6%), and Loss of control (2.0%). This pattern suggests that Preoccupation with food may be more sensitive to sociodemographic variation, possibly reflecting more cognitively mediated or socially influenced processes [10,11,32,39], whereas Loss of control may be more trait-like or neurobiologically anchored [5,9,10,11,32,38], and therefore less variable across demographic contexts.

The results help contextualize how reward-based eating may differ across populations and demographic subgroups. Italian participants reported significantly higher RED-13 total scores and Lack of Satiety scores than their Portuguese counterparts, converging with the latent model results that also found elevated latent means across RED dimensions in the Italian group. These findings may reflect cultural or dietary factors influencing perceptions of fullness or hedonic responsiveness, which are shaped by differences in traditional eating patterns, availability of palatable foods, and psychosocial factors related to emotional eating and food attitudes [7,8,39,40,41,42]. Variations in access to highly rewarding, energy-dense foods and cultural norms around eating occasions could contribute to elevated reward-based eating drive observed in the Italian sample compared to the Portuguese group.

The Polish results warrant particular attention and illustrate the importance of using complementary analytic methods. In the multivariate analysis based on observed scores, no significant differences were observed between students and the general population, and Polish participants scored significantly lower than Portuguese participants on the Lack of satiety subscale. In contrast, the latent mean comparisons revealed a more differentiated picture: compared to the reference group (Portuguese general population), Polish students showed significantly higher scores across all RED-13 dimensions and on the RED-X5, while the Polish general population presented a distinct profile, characterized by higher Loss of control but lower Lack of satiety and Preoccupation with food. This divergence may reflect both the added sensitivity of latent mean modeling [18,19,24] and the differing comparison structures used in each approach. While the multivariate analysis treated country and participant group (students vs. general population) as separate factors, the latent mean analysis compared five distinct groups, by separating students and the general population within countries.

Age showed the most consistent and pronounced effect across all multivariate models. Higher age was significantly associated with lower scores on the RED-13 total scale, all three subscales, and the RED-X5, with the strongest association observed for Preoccupation with food. This pattern aligns with evidence that both neurobiological sensitivity to reward and related behavioral tendencies decrease with age [33,34,35,36]. The stronger association with Preoccupation with food may additionally reflect age-related shifts in cognitive focus and emotion regulation [43,44], with older adults experiencing less intrusive food-related thoughts and lower emotional reactivity to eating cues.

Sex also showed significant but differentiated effects on RED scores. Women scored higher than men on the RED-13 total scale, the RED-X5, and especially on the Preoccupation with food subscale. These results are consistent with prior research showing that women tend to report stronger food cravings greater cognitive focus on eating, and more frequent emotional or dysregulated eating patterns [37,45,46,47]. In contrast, men scored significantly higher on the Lack of Satiety subscale. This may be influenced by the types of cravings typically experienced: men are more likely to crave savoury foods, while women more often report cravings for sweet items [37]. However, the relationship between food cravings (and their intensity), consumption, and the perception of satiety is complex, as many factors that influence satiety (e.g., energy density, texture, oral processing) vary widely across foods [48,49,50,51]. Altogether, these findings suggest that while women tend to score higher in overall reward-based eating, specific aspects of this behavior may vary by sex, perhaps reflecting both biological and psychosocial influences.

### 4.5. Limitations and Strengths

This study has some limitations that should be acknowledged. The design did not include an assessment of test–retest reliability, which limits conclusions regarding the temporal stability of the RED scales. The samples, composed of convenience samples of both students and general population participants, may not fully represent the broader demographics of each country, potentially limiting generalizability. Moreover, the absence of more restrictive inclusion and exclusion criteria (e.g., eating disorders, bariatric surgery, pregnancy), may have introduced additional heterogeneity into the samples, which could affect the generalizability of the findings, particularly regarding the associations between RED scores and BMI. Additionally, the lack of full scalar invariance constrains the interpretation of latent mean differences across groups, warranting caution when comparing scores between populations.

Despite these limitations, the present study has several important strengths. The rigorous translation and cultural adaptation process across three European countries enhances the scales’ cross-cultural applicability and semantic equivalence, allowing trustworthy comparisons between countries. Students are a sub-population easier to reach for research purposes, although they may differ from the overall population in eating behaviour characteristics. The inclusion of both student and general population samples improves ecological validity and relevance across demographic groups. The application of advanced psychometric techniques, such as confirmatory factor analysis and measurement invariance testing, provides robust evidence supporting the structural validity of both RED-13 and RED-X5. Furthermore, evidence of convergent validity with theoretically related constructs across different countries and population groups strengthens the conceptual validity of the scales. Finally, the translation, cultural adaptation, and psychometric evaluation of both the full and brief versions in this study allow their availability, providing practical flexibility for researchers and clinicians according to their assessment needs.

## 5. Conclusions

This study successfully translated and culturally adapted the RED-13 and RED-X5 scales for use in Portugal, Poland and Italy, demonstrating good psychometric properties. The factorial structure was supported across countries and demographic groups (higher education students and general population), with partial scalar invariance enabling cautious latent mean comparisons. Both versions showed strong internal consistency and convergent validity with related constructs, namely BMI and food cravings. The strong association between RED-X5 and RED-13 total scores supports the use of the brief version as a reliable proxy for the full scale when detailed subscale information is not required. Notably, reward-based eating tendencies varied across sociodemographic characteristics, highlighting the multidimensional and culturally nuanced nature of these behaviours. Overall, the RED-13 and its short form RED-X5 are reliable and valid tools for assessing reward-driven eating in diverse European contexts, suitable for a variety of research and applied settings.

## Figures and Tables

**Table 1:** Participant characteristics by country and group.

	Sex (Females) n (%)	Age (Years) Mean (SD)	BMI (kg/m^2^) Median (P25, P75)
Portugal: Students [n = 252]	189 (75.0)	28.1 (9.9)	22.5 (20.5, 24.9)
Portugal: General population [n = 602]	454 (75.4)	46.6 (12.8)	23.8 (21.8, 27.0)
Poland: Students [n = 376]	236 (62.8)	22.8 (6.8)	22.7 (20.2, 25.1)
Poland: General population [n = 424]	186 (43.9)	41.5 (12.8)	25.9 (23.1, 28.7)
Italy: Students [n = 95]	66 (69.5)	23.9 (6.3)	21.5 (19.7, 23.7)
Italy: General population [n = 250]	159 (63.6)	31.9 (13.7)	22.3 (20.4, 24.8)

SD: standard deviation. BMI: body mass index.

**Table 2:** Model fit indices for RED-13 configural, metric, scalar, and partial scalar invariance models.

Model	Configural Invariance	Metric Invariance	Scalar Invariance	Partial Scalar Invariance
χ^2^ (df)	828.066 (295)	911.704 (335)	1635.978 (387)	1084.684 (369)
*P*	<0.001	<0.001	<0.001	<0.001
χ^2^/df	2.807	2.722	4.227	2.842
NFI	0.935	0.928	0.872	0.918
RFI	0.914	0.917	0.871	0.913
TLI	0.943	0.946	0.898	0.942
CFI	0.957	0.953	0.899	0.945
RMSEA [90%CI]	0.030 [0.028, 0.033]	0.029 [0.027, 0.032]	0.040 [0.038, 0.042]	0.030 [0.028, 0.033]

df: degrees of freedom.

**Table 3:** Latent mean comparisons for RED-13 across groups.

	Factor 1 Loss of Control			Factor 2 Lack of Satiety			Factor 3 Preoccupation with Food		
	Estimate	S.E.	*p*	Estimate	S.E.	*p*	Estimate	S.E.	*p*
Portugal: Students [n = 252]	0.039	0.072	0.589	0.033	0.066	0.611	0.084	0.077	0.277
Portugal: Gen. pop. [n = 602]	0 (ref.)			0 (ref.)			0 (ref.)		
Poland: Students [n = 376]	0.286	0.061	<0.001	0.173	0.059	0.003	0.416	0.068	<0.001
Poland: Gen. pop. [n = 424]	0.164	0.061	0.007	−0.219	0.052	<0.001	−0.171	0.059	0.004
Italy [n = 345]	0.230	0.064	<0.001	0.435	0.063	<0.001	0.252	0.071	<0.001

S.E.: standard error.

**Table 4:** Model fit indices for RED-X5 configural, metric, scalar, and partial scalar invariance models.

Model	Configural Invariance	Metric Invariance	Scalar Invariance	Partial Scalar Invariance
χ^2^ (df)	20.794 (10)	48.637 (26)	413.183 (46)	101.264 (39)
*P*	0.023	0.005	<0.001	<0.001
χ^2^/df	2.079	1.871	8.982	2.597
NFI	0.994	0.985	0.875	0.969
RFI	0.968	0.972	0.864	0.961
TLI	0.983	0.987	0.877	0.975
CFI	0.997	0.993	0.887	0.981
RMSEA [90%CI]	0.023 [0.008, 0.037]	0.021 [0.011, 0.030]	0.063 [0.058, 0.069]	0.028 [0.022, 0.035]

df: degrees of freedom.

**Table 5:** Latent mean comparisons for RED-X5 across groups.

	Estimate	S.E.	*p*
Portugal: Students [n = 252]	0.050	0.043	0.249
Portugal: Gen. population [n = 602]	0 (ref.)		
Poland: Students [n = 376]	0.163	0.040	<0.001
Poland: Gen. population [n = 424]	−0.046	0.035	0.185
Italy [n = 345]	0.105	0.041	0.009

S.E.: standard error.

**Table 6:** Internal consistency.

	RED-13 Total α	RED-13 F1 LC α	RED-13 F2 LS α	RED-13 F3 PF α	RED-X5 α
Portugal: Students [n = 252]	0.868	0.769	0.655	0.902	0.748
Portugal: Gen. population [n = 602]	0.906	0.821	0.712	0.911	0.808
Poland: Students [n = 376]	0.874	0.777	0.674	0.881	0.737
Poland: Gen. population [n = 424]	0.889	0.820	0.723	0.903	0.787
Italy: Students [n = 95]	0.897	0.793	0.701	0.918	0.811
Italy: Gen. population [n = 250]	0.880	0.789	0.697	0.902	0.749
Mean (SD)	1.48 (0.72)	1.98 (0.82)	1.20 (0.82)	0.95 (0.94)	1.32 (0.79)

F1 LC: Factor 1—Loss of control (6 items). F2 LS: Factor 2—Lack of satiety (3 items). F3 PF: Factor 3—Preoccupation with food (4 items). α: Cronbach’s alpha coefficient. SD: standard deviation.

**Table 7:** Associations of RED scores with BMI and food cravings.

Total Sample [n = 1999]	BMI (kg/m^2^)	Cravings: Savoury (1 to 100)	Cravings: Sweets (1 to 100)
	rs (*p*)	r (*p*)	r (*p*)
RED-13—Total	0.169 (<0.001)	0.315 (<0.001)	0.286 (<0.001)
RED-13—F1 LC	0.248 (<0.001)	0.286 (<0.001)	0.269 (<0.001)
RED-13—F2 LS	0.108 (<0.001)	0.242 (<0.001)	0.168 (<0.001)
RED-13—F3 PF	0.043 (0.053)	0.256 (<0.001)	0.254 (<0.001)
RED-X5	0.166 (<0.001)	0.278 (<0.001)	0.270 (<0.001)

BMI: body mass index. F1 LC: Factor 1—Loss of control. F2 LS: Factor 2—Lack of satiety. F3 PF: Factor 3—Preoccupation with food. r: Pearson’s correlation coefficient. rs: Spearman’s correlation coefficient.

**Table 8:** Effects of country, group, sex and age on RED-13 total scores.

	RED-13—Total		
	β	*p*	η_p_^2^
Corrected model		<0.001	0.055
Country (Ref.: Portugal)		0.009	0.005
- Poland	0.006	0.882	0.000
- Italy	0.137	0.004	0.004
Group (Ref.: Gen. population)		0.697	0.000
- Students	−0.016		
Sex (Ref.: Female)		0.045	0.002
- Male	−0.069		
Age (years)	−0.010	<0.001	0.026
Adjusted R^2^	0.053		

Analysis of covariance (ANCOVA), n = 1999. Portugal: n = 854, Poland: n = 800, Italy: n = 345, General population: n = 1276, Students: n = 723, Females: n = 1290, Males: n = 709. β: ANCOVA coefficients. η_p_^2^: partial eta-squared.

**Table 9:** Effects of country, group, sex and age on RED-13 dimensions.

	RED-13—F1 LC			RED-13—F2 LS			RED-13—F3 PF		
	β	*p*	η_p_^2^	β	*p*	η_p_^2^	β	*p*	η_p_^2^
Corrected model		<0.001	0.023		<0.001	0.058		<0.001	0.091
Country (Ref.: Portugal)		0.149	0.002		<0.001	0.028		0.291	0.001
- Poland	0.059	0.165	0.001	−0.137	0.001	0.005	0.032	0.505	0.000
- Italy	0.101	0.068	0.002	0.263	<0.001	0.012	0.096	0.116	0.001
Group (Ref.: Gen. pop.)		0.239	0.001		0.890	0.000		0.609	0.000
- Students	−0.055			0.006			0.026		
Sex (Ref.: Female)		0.646	0.000		0.026	0.002		<0.001	0.026
- Male	0.018			0.086			−0.314		
Age (years)	−0.008	<0.001	0.012	−0.009	<0.001	0.017	−0.014	<0.001	0.032
Adjusted R^2^	0.020			0.056			0.089		

Multivariate analysis of covariance (MANCOVA), n = 1999. Portugal: n = 854, Poland: n = 800, Italy: n = 345, General population: n = 1276, Students: n = 723, Females: n = 1290, Males: n = 709. F1 LC: Factor 1—Loss of control. F2 LS: Factor 2—Lack of satiety. F3 PF: Factor 3—Preoccupation with food. β: MANCOVA coefficients. η_p_^2^: partial eta-squared.

**Table 10:** Effects of country, group, sex and age on RED-X5 scores.

	RED-X5		
	β	*p*	η_p_^2^
Corrected model		<0.001	0.044
Country (Ref.: Portugal)		0.053	0.003
- Poland	−0.016	0.695	0.000
- Italy	0.106	0.045	0.002
Group (Ref.: Gen. population)		0.519	0.000
- Students	−0.029		
Sex (Ref.: Female)		<0.001	0.006
- Male	−0.128		
Age (years)	−0.010	<0.001	0.020
Adjusted R^2^	0.042		

Analysis of covariance (ANCOVA), n = 1999. Portugal: n = 854, Poland: n = 800, Italy: n = 345, General population: n = 1276, Students: n = 723, Females: n = 1290, Males: n = 709. β: ANCOVA coefficients. η_p_^2^: partial eta-squared.

## Data Availability

The data that support the findings of this study are available from the authors, upon reasonable request.

## Supplementary Materials

The following supporting information can be downloaded at: https://www.mdpi.com/article/10.3390/nu18010049/s1, Table S1: Final versions of the RED-13 in European Portuguese, Polish and Italian. Table S2: Associations of RED scores with BMI and food cravings, stratified by country, group, and sex.
