# Supplementary material for: Cross-Cultural Adaptation and Psychometric Properties of the Reward-Based Eating Drive Scale (RED-13) and Its Brief Version (RED-5X) in Three European Countries
Source: Nutrients. 2025 Dec 23;18(1):49. doi: 10.3390/nu18010049 (PMC12788072; doi:10.3390/nu18010049)
Supplement: Supplementary file 1 [file nutrients-18-00049-s001.zip › Supplementary_material_1.pdf]

Table S1. Final versions of the RED-13 in European Portuguese, Polish and Italian

European Portuguese	Polish	Italian
Escala do Impulso para Comer Baseado na Recompensa	Skala skłonności do nagradzania się jedzeniem	Scala RED (Reward Based Eating Drive)
Por favor, leia cada afirmação e indique o quanto concorda ou discorda.<br>0 Discordo fortemente<br>1 Discordo<br>2 Não concordo nem discordo<br>3 Concordo<br>4 Concordo fortemente	Proszę przeczytaj każde stwierdzenie i wskaż, w jakim stopniu się z nim zgadzasz lub nie zgadzasz.<br>0 Zdecydowanie się nie zgadzam<br>1 Nie zgadzam się<br>2 Ani się zgadzam, ani się nie zgadzam<br>3 Zgadzam się<br>4 Zdecydowanie się zgadzam	Si prega di leggere ogni frasi e di indicare il livello di accordo o disaccordo.<br>0 Fortemente in disaccordo<br>1 In disaccordo<br>2 Nè in accordo nè in disaccordo<br>3 D'accordo<br>4 Fortemente d'accordo
1. Sinto-me descontrolada/o na presença de comida deliciosa.	1. Nie mogę się opanować mając przed sobą pyszne jedzenie.	1. Sento di perdere il controllo in presenza di cibi deliziosi.
2. Quando começo a comer, parece que não consigo parar.	2. Gdy zacznę jeść, po prostu nie mogę przestać.	2. Quando inizio a mangiare, sento di non riuscire a fermarmi.
3. É difícil para mim deixar comida no meu prato.	3. Trudno mi zostawić jedzenie na talerzu.	3. Trovo difficile lasciare del cibo nel piatto.
4. Quando se trata de alimentos que eu adoro, não tenho força de vontade.	4. Nie mam silnej woli, jeśli chodzi o jedzenie, które uwielbiam.	4. Quando si tratta di cibi che amo, non ho alcuna forza di volontà.
5. Fico com tanta fome que o meu estômago costuma parecer um poço sem fundo.	5. Dopada mnie taki głód, że mój żołądek często wydaje się być studnią bez dna.	5. Spesso, ho una fame così intensa da sembrare che il mio stomaco non abbia fondo.
6. Não fico cheia/o facilmente.	6. Trudno mi najeść się do syta.	6. Non mi sento pieno facilmente.
7. Parece que na maioria do tempo que passo acordada/o estou preocupada/o com pensamentos sobre comer ou não comer.	7. Wygląda na to, że przez większość czasu pochłaniają mnie myśli o jedzeniu lub niejedzeniu.	7. Mi sembra che la maggior parte delle mie ore di veglia siano occupate da pensieri sul cibo, sulla sua assunzione o meno.
8. Há dias em que parece que não consigo pensar em mais nada para além de comida.	8. Miewam dni, kiedy nie mogę myśleć o niczym innym poza jedzeniem.	8. Ci sono giorni in cui non riesco a pensare ad altro che al cibo.
9. A comida está sempre nos meus pensamentos.	9. Ciągle myślę o jedzeniu.	9. Il cibo è sempre nei miei pensieri.
10. Sinto-me sempre com fome.	10. Cały czas czuję głód.	10. Sento sempre fame.
11. Não consigo parar de pensar em comer, por mais que eu tente.	11. Nie mogę przestać myśleć o jedzeniu, bez względu na to, jak bardzo się staram.	11. Non riesco a smettere di pensare al cibo, non importa quanto ci provi.
12. Dou por mim a continuar a comer certos alimentos, mesmo que já não tenha fome.	12. Kontynuuję jedzenie pewnych produktów spożywczych, mimo że nie odczuwam już głodu.	12. Continuo a mangiare certi cibi anche quando non ho più fame.
13. Se a comida me sabe bem, eu como mais do que o habitual.	13. Jeśli jedzenie mi smakuje, jem więcej niż zwykle.	13. Se il cibo mi piace, mangio più del solito.
