# Supplementary material for: Cross-Cultural Adaptation and Psychometric Properties of the Reward-Based Eating Drive Scale (RED-13) and Its Brief Version (RED-5X) in Three European Countries
Source: Nutrients. 2025 Dec 23;18(1):49. doi: 10.3390/nu18010049 (PMC12788072; doi:10.3390/nu18010049)
Supplement: Supplementary file 1 [file nutrients-18-00049-s001.zip › Supplementary_material_2.pdf]

Table S2. Associations of RED scores with BMI and food cravings, stratified by country, group, and sex

PORTUGAL Students	Females *[n = 189]*			Males *[n = 63]*		
	**BMI rs (p)**	**Cravings: savoury r (p)**	**Cravings: sweets r (p)**	**BMI rs (p)**	**Cravings: savoury r (p)**	**Cravings: sweets r (p)**
**RED-13 - Total**	0.330 (< 0.001)	0.216 (0.003)	0.257 (< 0.001)	0.088 (0.494)	0.444 (< 0.001)	0.134 (0.293)
**RED-13 - F1 LC**	0.357 (< 0.001)	0.235 (0.001)	0.251 (0.001)	0.073 (0.568)	0.384 (0.002)	0.100 (0.433)
**RED-13 - F2 LS**	0.231 (0.001)	0.127 (0.083)	0.112 (0.125)	-0.066 (0.608)	0.275 (0.029)	0.007 (0.958)
**RED-13 - F3 PF**	0.205 (0.005)	0.145 (0.046)	0.233 (0.001)	0.094 (0.464)	0.322 (0.010)	0.167 (0.190)
**RED-X5**	0.318 (< 0.001)	0.192 (0.008)	0.252 (< 0.001)	0.109 (0.393)	0.455 (< 0.001)	0.144 (0.261)
**PORTUGAL General population**	**Females** *[n = 454]*			**Males** *[n = 148]*		
	**BMI rs (p)**	**Cravings: savoury r (p)**	**Cravings: sweets r (p)**	**BMI rs (p)**	**Cravings: savoury r (p)**	**Cravings: sweets r (p)**
**RED-13 - Total**	0.395 (< 0.001)	0.430 (< 0.001)	0.403 (< 0.001)	0.294 (< 0.001)	0.363 (< 0.001)	0.404 (< 0.001)
**RED-13 - F1 LC**	0.424 (< 0.001)	0.423 (< 0.001)	0.396 (< 0.001)	0.291 (< 0.001)	0.352 (< 0.001)	0.361 (< 0.001)
**RED-13 - F2 LS**	0.275 (< 0.001)	0.382 (< 0.001)	0.312 (< 0.001)	0.252 (0.002)	0.197 (0.016)	0.310 (< 0.001)
**RED-13 - F3 PF**	0.322 (< 0.001)	0.309 (< 0.001)	0.321 (< 0.001)	0.216 (0.009)	0.329 (< 0.001)	0.348 (< 0.001)
**RED-X5**	0.380 (< 0.001)	0.378 (< 0.001)	0.369 (< 0.001)	0.327 (< 0.001)	0.323 (< 0.001)	0.344 (< 0.001)
**POLAND Students**	**Females** *[n = 236]*			**Males** *[n = 140]*		
	**BMI rs (p)**	**Cravings: savoury r (p)**	**Cravings: sweets r (p)**	**BMI rs (p)**	**Cravings: savoury r (p)**	**Cravings: sweets r (p)**
**RED-13 - Total**	0.036 (0.587)	0.247 (< 0.001)	0.182 (0.005)	0.086 (0.314)	0.223 (0.008)	0.207 (0.014)
**RED-13 - F1 LC**	0.121 (0.063)	0.236 (< 0.001)	0.170 (0.009)	0.063 (0.460)	0.208 (0.014)	0.210 (0.013)
**RED-13 - F2 LS**	-0.034 (0.605)	0.166 (0.011)	0.108 (0.098)	0.058 (0.495)	0.132 (0.121)	0.083 (0.329)
**RED-13 - F3 PF**	-0.029 (0.658)	0.200 (0.002)	0.161 (0.013)	0.108 (0.205)	0.197 (0.020)	0.189 (0.025)
**RED-X5**	0.044 (0.498)	0.185 (0.004)	0.149 (0.022)	0.062 (0.467)	0.212 (0.012)	0.264 (0.002)
**POLAND General population**	**Females** *[n = 186]*			**Males** *[n = 238]*		
	**BMI rs (p)**	**Cravings: savoury r (p)**	**Cravings: sweets r (p)**	**BMI rs (p)**	**Cravings: savoury r (p)**	**Cravings: sweets r (p)**
**RED-13 - Total**	0.240 (0.001)	0.270 (< 0.001)	0.313 (< 0.001)	0.323 (< 0.001)	0.136 (0.036)	0.202 (0.002)
**RED-13 - F1 LC**	0.289 (< 0.001)	0.197 (0.007)	0.288 (< 0.001)	0.335 (< 0.001)	0.165 (0.011)	0.249 (< 0.001)
**RED-13 - F2 LS**	0.199 (0.006)	0.207 (0.005)	0.199 (0.007)	0.256 (< 0.001)	0.070 (0.279)	0.131 (0.044)
**RED-13 - F3 PF**	0.073 (0.320)	0.312 (< 0.001)	0.299 (< 0.001)	0.131 (0.044)	0.047 (0.470)	0.043 (0.513)
**RED-X5**	0.234 (0.001)	0.297 (< 0.001)	0.338 (< 0.001)	0.301 (< 0.001)	0.110 (0.089)	0.144 (0.026)
**ITALY Students**	**Females** *[n = 66]*			**Males** *[n = 29]*		
	**BMI rs (p)**	**Cravings: savoury r (p)**	**Cravings: sweets r (p)**	**BMI rs (p)**	**Cravings: savoury r (p)**	**Cravings: sweets r (p)**
**RED-13 - Total**	0.296 (0.016)	0.205 (0.100)	0.325 (0.008)	0.189 (0.326)	0.472 (0.010)	0.409 (0.028)
**RED-13 - F1 LC**	0.280 (0.023)	0.132 (0.291)	0.262 (0.034)	0.130 (0.501)	0.481 (0.008)	0.355 (0.059)
**RED-13 - F2 LS**	0.326 (0.008)	0.150 (0.229)	0.121 (0.333)	0.284 (0.135)	0.384 (0.039)	0.285 (0.135)
**RED-13 - F3 PF**	0.214 (0.085)	0.226 (0.068)	0.371 (0.002)	0.256 (0.180)	0.370 (0.048)	0.422 (0.023)
**RED-X5**	0.285 (0.020)	0.210 (0.091)	0.345 (0.005)	0.284 (0.135)	0.465 (0.011)	0.427 (0.021)

ITALY General population	Females *[n = 159]*			Males *[n = 91]*		
	**BMI**	**Cravings: savoury**	**Cravings: sweets**	**BMI**	**Cravings: savoury**	**Cravings: sweets**
	**rs (p)**	**r (p)**	**r (p)**	**rs (p)**	**r (p)**	**r (p)**
**RED-13 - Total**	0.293 (< 0.001)	0.291 (< 0.001)	0.189 (0.017)	0.171 (0.104)	0.160 (0.130)	0.232 (0.027)
**RED-13 - F1 LC**	0.357 (< 0.001)	0.193 (0.015)	0.134 (0.093)	0.305 (0.003)	0.021 (0.842)	0.160 (0.131)
**RED-13 - F2 LS**	0.245 (0.002)	0.235 (0.003)	0.067 (0.399)	0.015 (0.889)	0.215 (0.040)	0.175 (0.098)
**RED-13 - F3 PF**	0.132 (0.096)	0.322 (< 0.001)	0.254 (0.001)	0.027 (0.799)	0.194 (0.065)	0.228 (0.030)
**RED-X5**	0.291 (< 0.001)	0.278 (< 0.001)	0.178 (0.025)	0.210 (0.045)	0.104 (0.326)	0.156 (0.140)

BMI: body mass index. F1 LC: Factor 1 - Loss of control. F2 LS: Factor 2 - Lack of satiety. F3 PF: Factor 3 - Preoccupation with food. r: Pearson's correlation coefficient. rs: Spearman's correlation coefficient.
